# Clinical utility of computed tomography Hounsfield characterization for percutaneous nephrolithotomy: a cross-sectional study

**DOI:** 10.1186/s12894-017-0296-1

**Published:** 2017-11-16

**Authors:** Andrea Gallioli, Elisa De Lorenzis, Luca Boeri, Maurizio Delor, Stefano Paolo Zanetti, Fabrizio Longo, Alberto Trinchieri, Emanuele Montanari

**Affiliations:** 1 Fondazione IRCCS Ca’ Granda Ospedale Maggiore Policlinico, Department of Urology, University of Milan, Via della Commenda 15, 20122 Milan, Italy; 2 Istituto Europeo di Oncologia, Department of Urology, University of Milan, Via Giuseppe Ripamonti 435, 20141 Milan, Italy; 3 Department of Urology, Ospedale Alessandro Manzoni Lecco, Via dell’Eremo 9/11, 23900 Lecco, Italy

**Keywords:** Hounsfield, Kidney stone, Percutaneous nephrolithotomy, Computed tomography

## Abstract

**Background:**

Computed Tomography (CT) is considered the gold-standard for the pre-operative evaluation of urolithiasis. However, no Hounsfield (HU) variable capable of differentiating stone types has been clearly identified. The aim of this study is to assess the predictive value of HU parameters on CT for determining stone composition and outcomes in percutaneous nephrolithotomy (PCNL).

**Methods:**

Seventy seven consecutive cases of PCNL between 2011 and 2016 were divided into 4 groups: 40 (52%) calcium, 26 (34%) uric acid, 5 (6%) struvite and 6 (8%) cystine stones. All images were reviewed by a single urologist using abdomen/bone windows to evaluate: stone volume, core (HUC), periphery HU and their absolute difference. HU density (HUD) was defined as the ratio between mean HU and the stone’s largest diameter. ROC curves assessed the predictive power of HU for determining stone composition/stone-free rate (SFR).

**Results:**

No differences were found based on the viewing window (abdomen vs bone). Struvite stones had values halfway between hyperdense (calcium) and low-density (cystine/uric acid) calculi for all parameters except HUD, which was the lowest. All HU variables for medium-high density stones were greater than low-density stones (*p* < 0.001). HUC differentiated the two groups (cut-off 825 HU; specificity 90.6%, sensitivity 88.9%). HUD distinguished calcium from struvite (mean ± SD 51 ± 16 and 28 ± 12 respectively; *p* = 0.02) with high sensitivity (82.5%) and specificity (80%) at a cut-off of 35 HU/mm. Multivariate analysis revealed HUD ≥ 38.5 HU/mm to be an independent predictor of SFR (OR = 3.1, *p* = 0.03). No relationship was found between HU values and complication rate.

**Conclusions:**

HU parameters help predict stone composition to select patients for oral chemolysis. HUD is an independent predictor of residual fragments after PCNL and may be fundamental to categorize it, driving the imaging choice at follow-up.

**Electronic supplementary material:**

The online version of this article (10.1186/s12894-017-0296-1) contains supplementary material, which is available to authorized users.

## Background

Computed Tomography (CT) is the gold standard for the pre-operative study of stones and influences the choice of surgical strategy [1]. Hounsfield Units (HU) indicate the hardness of renal calculi and identify high density stones to be excluded from shockwave lithotripsy (SWL) [2]. Several in vitro studies have demonstrated the utility of CT in predicting stone composition [3–6]. The use of different HU parameters, such as Hounsfield Density (HUD), has been proposed to distinguish stone groups in vivo [7–12]. In order to further improve the differentiation of stones, scans conducted with the bone window setting have been attempted [13] and, recently, the mean HU value has been suggested as a predictor for the stone-free rate (SFR) after percutaneous nephrolithotomy (PCNL) [14]. To the best of our knowledge, no study has evaluated the role of a wide range of HU parameters in vivo. The aim of this study is to evaluate the clinical significance and utility of the HU parameters determined during pre-operative CT study, using bone and soft tissue window (=abdomen), to be predictive factors of stone composition, SFR and complication rate in a cohort of patients submitted to PCNL.

## Patients and methods

We retrospectively reviewed the institutional stone registry between January 2011 and April 2016. 284 patients submitted to PCNL were found. Inclusion criteria were: I) the availability of a pre-operative CT-scan, II) a maximum stone diameter > 4 mm, III) the availability at our Institution of the biochemical analysis of the stones, IV) a prominent stone component >50% in mixed stones. Patients with pre-operative urinary stents were excluded.

**Fig. 1:**
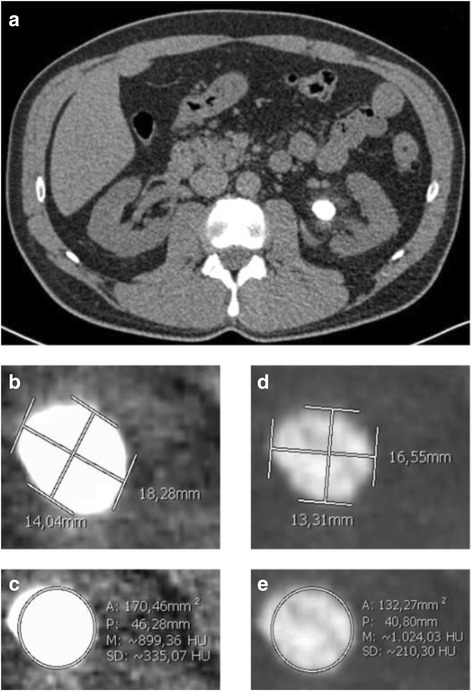
Calculus of left renal pelvis (**a**). Maximum/perpendicular diameters and ROI of the stone on soft tissue (**b**, **c**) and bone (**d**, **e**) window scans. Legend: A = area; P = perimeter; M = mean Hounsfield; SD = standard deviation

CT scans were performed with a 64-detector row Lightspeed VCT scanner (General Electric Healthcare, Milwaukee, WI) with tube voltage 120 kV, energy >100 mA, pitch 1:1, slice thickness comprised between 0.6 and 5 mm. CT scans were evaluated by the same Urologist (M.D.), blinded to the stone composition, using PACS Synapse Fujifilm version 4.0 software at 4× zoom. Both bone (X_B_) and soft tissue (X_ST_) windows were analyzed. The slice with the stone’s largest diameter (D_1_) on the axial plane was selected and the following variables were recorded: perpendicular diameter (D_2_), HU value at the center of the stone (HUC) and the mean HU value (HUM) which was calculated by generating an extensive circular Region Of Interest (ROI). The HU value at the stone’s periphery (HUP) was obtained from the mean of HU values at extremities of D_1_ and D_2_ (Fig. 1). HUD was defined as the ratio between HUM and D_1_. The absolute HU difference between the stone center and periphery was calculated as HUC minus HUP (∆HU). The stone’s Area (A) and Volume (V) were estimated using Tiselius formulas [15]. D_3_ was determined as the maximum diameter at coronal plane CT scans.

PCNL was performed by the same surgeon (E.M.) for all cases. The procedure was conducted in the supine position. An open-ended ureteral catheter was positioned before an ultrasound-guided renal puncture. A one-shot dilatation of the percutaneous access tract was conducted using an Amplatz 24 Charrier dilator [16]. Lithotripsy was performed with ballistic, ultrasound or holmium laser energy while fragments were removed with an endoscopic nipper or basket. An 8 Ch nephrostomy was positioned at the conclusion of each procedure.

The evaluated peri-operative parameters were surgical time (from the access puncture to nephrostomy), reduction of hemoglobin, fever, need for transfusion and hospital stay. Complications were classified using the Clavien-Dindo score modified by the Clinical Research Office of Endourological Society (CROES) and divided in three groups: no complications, slight complications (Clavien-Dindo 1/2), severe complications (Clavien-Dindo ≥3) [17].

SFR, defined as the absence of residual fragments, was assessed by US or CT after 3 months. Stone composition was determined by infrared spectrophotometry (Thermo Scientific Nicolet™ iS™ 10) and stones were categorized into 4 groups: 40 (52%) calcium, 26 (34%) uric acid, 6 (8%) cystine and 5 (6%) struvite. The patient group was composed of 52 (67%) men and 25 (33%) women with a mean age of 57 (range 14–92) years. No differences in terms of sex and age were found between stone composition groups, with the exception of patients with cystine stones who had a younger average age (32 years) (Additional file 1: Table S1).

Statistical analysis was performed utilizing GraphPad Prism v 5 (GraphPad Software Inc., California, USA) and SPSS v 13.0 (IBM Cor., Armonk, NY, USA). T-tests and one-way analysis of variance (ANOVA) tests were used for group comparisons. Chi-Square and logistic regressions were calculated for categorical parameters. Receiver Operating Characteristic (ROC) curves were generated to find HU value cut-offs (defined as Youden J Index) to predict stone composition and SFR. Statistically significant differences were assumed for *p* values less than 0.05.

## Results

Analysis of the groups showed that stone dimensions appeared generally larger using the CT soft tissue window, but no significant differences in D_1_, A and V were observed. On the contrary, HU values were higher using the CT bone window (Table 1).

**Table 1:** Stone characteristics on soft tissue/bone window CT (mean ± SD)

	Soft tissue (Bone) windows	p	Calcium	Uric acid	Cystine	Struvite
D_1_ (mm)	19 ± 7 (17 ± 7)	0.34	18 ± 6 (16 ± 6)	19 ± 8 (18 ± 8)	18 ± 8 (17 ± 8)	24 ± 6 (23 ± 6)
Area (mm^2^)	188 ± 137 (164 ± 126)	0.26	174 ± 122 (148 ± 111)	190 ± 150 (168 ± 137)	204 ± 168 (185 ± 160)	275 ± 152 (250 ± 143)
Volume (mm^3^)	2914 ± 3481 (2438 ± 3084)	0.37	2495 ± 3072 (2024 ± 2669)	3097 ± 4034 (2612 ± 3563)	4178 ± 4228 (3714 ± 3964)	3792 ± 2964 (3318 ± 2637)
HUC	942 ± 378 (986 ± 389)	0.48	1190 ± 251 (1240 ± 259)	606 ± 276 (638 ± 271)	683 ± 75 (708 ± 66)	1010 ± 394 (1090 ± 439)
HUP	314 ± 55 (395 ± 93)	*<0.001*	*330 ± 59 (426 ± 85)* ^a^	*287 ± 41 (358 ± 98)* ^a^	300 ± 45 (358 ± 49)	340 ± 42 (390 ± 96)
HUM	687 ± 265 (761 ± 306)	0.11	835 ± 233 (941 ± 270)	500 ± 203 (531 ± 216)	542 ± 86 (608 ± 86)	650 ± 235 (700 ± 255)
∆HU	628 ± 356 (590 ± 347)	0.5	860 ± 234 (814 ± 245)	319 ± 266 (280 ± 216)	383 ± 75 (350 ± 45)	670 ± 411 (700 ± 434)
HUD	41 ± 19 (50 ± 26)	*0.01*	*51 ± 16 (63 ± 22)* ^a^	31 ± 18 (36 ± 22)	35 ± 14 (43 ± 21)	28 ± 12 (33 ± 16)

^a^ Significant difference (*p* ≤ 0.01) between HU values at soft tissue versus bone windows

Legend: D_1_ = stone’s largest diameter at axial plane; HUC = HU at the center; HUP = HU value at periphery; HUM = HU mean value; ∆HU = HUC-HUP; HUD = ratio between HUM and D_1_

**Fig. 2:**
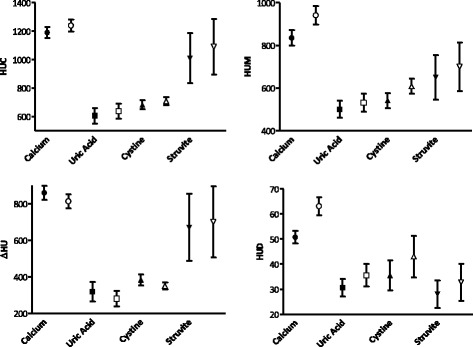
Visual distribution of HUC (1a), HUM (1b), ∆HU (1c), HUD (1d) evaluated in soft tissue (●) and bone (○) scans for all stone types. Legend: HUC = HU at the center of the stone; HUM = HU mean value; ∆HU = difference between HUC and HU at stone’s periphery; HUD = ratio between HUM and stone’s largest diameter at axial plane

Comparisons were made between the stone groups on the results obtained for 4 variables (HUM, ∆HU, HUC, HUD) using both the bone and soft tissue windows. Calcium stones were consistently hyperdense while uric acid and cystine stones had lower HU values. Struvite had intermediate values on all variables with the exception of HUD, which was the lowest among all stone groups (Fig. 2).

The mean values for calcium stones differed from those of the uric acid calculi on every parameter considered while cystine stones did not differentiate from struvite/uric acid calculi.

The values for HUC, HUM and ∆HU, but not HUD, differed between calcium and cystine stones. Struvite calculi had significantly higher values than uric acid stones for HUC and ∆HU, with ∆HU_B_ being the parameter that best differentiated them (mean ± SD 700 ± 434 vs 280 ± 216; *p* = 0.004).

Calcium and struvite stones differed significantly for HUD_ST_ (mean ± SD 51 ± 16 and 28 ± 12 respectively; *p* = 0.02). For a HUD_ST_ cut-off of 35 HU/mm, sensitivity was 82.5%, specificity 80%, negative predictive value (NPV) 36% and positive predictive value (PPV) 97%.

In order to apply these results in a clinical setting we grouped hypodense stones (uric acid, cystine) and compared them with hyperdense stones (calcium, struvite), finding that the hypodense had statistically lower HU values (*p* < 0.001). HUC_ST_ more accurately differentiated the two groups with a specificity of 90.6%, sensitivity 88.9%, PPV 93% and NPV 85.3% at a cut-off of 825 HU (Additional file 2: Figure S1).

Mean surgical time was 123 (40–240) minutes, medium hospital stay was 6 (1–15) days. Linear regression revealed that hospital stay was inversely correlated with HUD_ST_ (*p* = 0.04) and HUD_B_ (*p* = 0.02).

In 42 (55%) patients no complications were recorded, in 24 (32%) and 11 (13%) Clavien-Dindo grade 1–2 and grade ≥ 3 complications were observed, respectively. A hemoglobin drop necessitating blood transfusion was observed in 6 (7.8%) cases. HUD values in patients requiring transfusion, irrespective of the window used, were generally higher (mean HUD_ST_ 54.89 ± 10.58 vs. 40.13 ± 2.14, *p* = 0.06; mean HUD_B_ 69.78 ± 14.63 vs 48.6 ± 2.86, *p* = 0.05). The other evaluated peri-operative data were not related to HU values. SFR was 61% with no differences according to stone composition (*p* = 0.37). However, SFR was significantly higher in patients with stones <2 cm compared to those with stones deemed to be ≥2 cm using soft tissue (71% vs 40%; *p* < 0.009) and bone scans (70% vs 42%; *p* < 0.01). To evaluate whether an HU parameter was predictive of SFR we analyzed each variable, generating ROC curves. HUD_ST_ (cut-off 38.5 HU/mm) was the best SFR predictive factor (AUC 0.66, sensitivity 70%, specificity 63.8%, OR 4.12, *p* = 0.005; Table 2). Multivariate analysis revealed HUD_ST_ to be a significant predictor of SFR regardless of stone diameter (OR = 3.1, *p* = 0.03).

**Table 2:** Specificity and sensitivity of HUD in predicting stone-free rate at 3 months and relative univariate analysis

		ROC curve				Stone free		Univariate analysis		
	Cut-off	AUC	Sens	Spec	p	no (%)	yes (%)	OR	(95%- CI)	p
HUD_ST_	<38.5					21 (55)	17 (45)	1.00 Ref		
	≥38.5	0.66	70%	63.8%	*0.016*	9 (23)	30 (77)	4.12	(1.54–10.99)	*0.005*
HUD_B_	<49					21 (54)	18 (46)	1.00 Ref		
	≥49	0.67	70%	61%	*0.015*	9 (24)	29 (76)	3.76	(1.42–9.99)	*0.008*

Legend: *HUD* ratio between HU mean value and stone’s largest diameter at axial plane on soft tissue (ST) or bone (B) CT window, *OR* Odds ratio, *CI* Confidence interval

## Discussion

The current study evaluates the clinical applications of HU characterization using bone and soft tissue window CT scans in a cohort of patients treated with PCNL.

At present, there is no proven method to differentiate stone types prior to endosurgery or SWL. However, the treatment choice for intrarenal stones is based on stone dimension, location and HUM [18].

Mostafavi et al. has shown the predictive value of HU obtained from CT scans to differentiate uric acid, struvite and calcium oxalate kidney stones, while Dual-Energy CT has been proposed, albeit with controversial results, to improve HU power to predict stone composition [5, 9, 19]. However, this procedure is impractical as it is not available in the majority of the hospitals. 

Eisner et al. explored the precision of bone and abdomen window scans in measuring ureteral stones that were then spontaneously passed and physically measured. They concluded that the bone window offers a substantially more accurate estimate than the abdomen window [13]. Our study shows that stone diameter is smaller and HU values are higher when observed with the bone window, probably due to the better contrast provided by this window. However, such differences are clinically irrelevant.

Torricelli et al. analyzed the predictive power of HUC to differentiate uric acid (*n* = 47), calcium oxalate (*n* = 36) and cystine (*n* = 30) stones and concluded that HUC of calcium oxalate stones is significantly higher than that of uric acid stones [11]. Two studies have explored the possibility of dividing the HU value by the stone’s maximum diameter to reduce the bias derived from the observation that the bigger the stone, the higher the HU, regardless of the type of calculi. Nakada et al. reported the utility of the maximum HU/size ratio in discriminating uric acid versus calcium stones (28.8 ± 27.2 vs 49.1 ± 85.2; *p* = 0.0001) in a cohort of 99 patients [7].

Motley et al. confirmed the utility of the HU/size ratio (HUD), showing that it discriminated calcium (*n* = 87; mean ± SD = 105 ± 43), uric acid (*n* = 7; 50 ± 24), cystine (*n* = 2; 45 ± 4) and struvite (*n* = 4; 53 ± 28) stones [8].

Our study further confirms the capability of HU parameters to differentiate uric acid and cystine stones from calcium stones, regardless of the type of HU variable analyzed. Uric acid calculi have lower ∆HU and HUC values than struvite stones, while cystine calculi have HU values similar to stones composed of uric acid and struvite. However, the diagnosis of cystine stones is also guided by laboratory and epidemiological data (Brand’s test, urinary pH, crystals, uricosuria, uricemia, patient age).

HU measurements are useful if they first provide differentiation of medium-high (calcium, struvite) from low dense (uric acid, cystine) stones and then distinguish calcium from struvite stones. HUC_ST_ is the best predictor of stone density at a cut-off of 825 HU with a PPV of 93% and a NPV of 85.3%. Our study also demonstrates that struvite has low HUD, which differentiates it from calcium with a high sensitivity (82.5%) and specificity (80%).

**Fig. 3:**
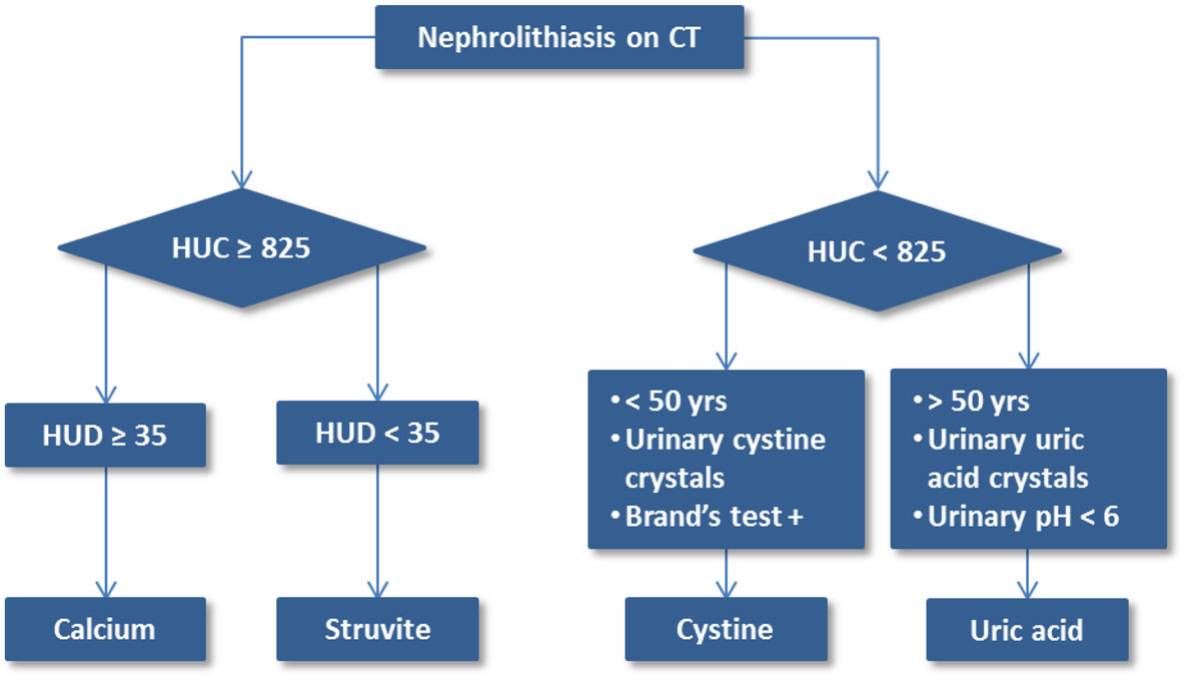
Stone composition assessment using HU values on CT. Legend: HUC = HU at the center of the stone; HUD = ratio between HU mean value and stone’s largest diameter at axial plane

These results suggest the utility of creating a flow-chart based on HUC and HUD values with integrated laboratory and demographic data to pre-operatively recognize stone composition (Fig. 3). The possibility to characterize uric acid stones with such accuracy may help to individuate candidates for oral chemolysis.

HU measurements are also known to be predictive factors of SFR after PCNL as described by CROES in a cohort of 5803 patients. Gücük et al. demonstrated that a HUM lower than 677.5 HU is a predictor of PCNL failure, even if the AUC was only 0.299 [14, 20]. In our study HUD_ST_ is an independent predictor of PCNL failure when lower than 38.5 HU/mm, indicating a 3.1 folds higher risk of residual stones. This may be due to the low sensitivity of intra-operative fluoroscopy in detecting stones less dense than 500 HU as reported by Chua et al. [21]. Furthermore, low HUD also indicates a large stone, which is one of the best-known predictive factors of PCNL failure. HUD, evaluated with other data such as the number of stones, their dimensions and the intraoperative stone clearance impression, can help categorize the risk of residual fragments. For high risk cases, a non-contrast CT should be requested at follow-up; otherwise an abdominal ultrasound might be preferred.

HU values were also correlated with peri-operative data. Our study shows that the higher the HUD, the shorter the hospitalization time. This result is in line with generally lower complication rates for hyperdense calculi, as described by CROES [20]. In contrast, the need for transfusion was directly related to HUD values, consistent with the data of Gücük et al. who reported a larger decrease in hemoglobin in cases of high HUM [14]. This can be explained by the energy needed to break hard stones into fragments, which can increase the risk of mucosal damage.

Our study has some limitations. First, it is a retrospective study with relatively few patients. We did not differentiate between calcium oxalate monohydrate and dihydrate stones because of the limited number of patients. Furthermore, the CT collimation varied between 0.6 and 5 mm; however, we tried to reduce this limit by only evaluating stones larger than 4 mm to increase the precision of each procedure.

For these reasons perspective studies should be conducted to confirm the results we describe.

## Conclusions

HU measurements at CT scan may help predict stone composition, regardless of the window setting (i.e. bone or abdomen) used. HUC accurately differentiates medium to high (struvite, calcium) from low (uric acid, cystine) density stones, a factor that can aid in selecting patients for oral chemolysis, while HUD distinguishes struvite from calcium calculi. Of note, HUD is an independent predictor of stone-free status after PCNL at three-month follow-up. Thus, it may be a useful tool for categorizing the risk of residual fragments and planning imaging follow-up.

## Additional files

Additional file 1: Table S1. Patients’ epidemiologic and stone characteristics. (DOCX 13 kb)

Additional file 2: Figure S1. ROC curve of HUC on soft tissue window CT scan (HUC_ST_) to discriminate between hypodense and hyperdense stones. Legend: HUC = HU at the center of the stone. (DOCX 16 kb)

## Abbreviations

∆HU: difference between HUC and HUP
A: area
D_1_: stone’s largest diameter at axial plane
D_2_: D_1_ perpendicular diameter
D_3_: maximum diameter at coronal plane
HU: Hounsfield units
HUC: HU value at the center of the stone
HUD: ratio between HUM and D_1_
HUM: HU mean value
HUP: HU value at stone’s periphery
PCNL: percutaneous nephrolithotomy
SFR: stone-free rate
SWL: shockwave lithotripsy
V: volume
X_B_: bone window scans
X_ST_: soft tissue window scans

## References

[1] Turk C, Petrik A, Sarica K (2016). EAU guidelines on diagnosis and conservative Management of Urolithiasis. Eur Urol.

[2] Hounsfield GN (1973). Computerized transverse axial scanning (tomography). 1. Description of system. Br J Radiol.

[3] Mitcheson HD, Zamenhof RG, Bankoff MS, Prien EL (1983). Determination of the chemical composition of urinary calculi by computerized tomography. J Urol.

[4] Newhouse JH, Prien EL, Amis ES (1984). Computed tomographic analysis of urinary calculi. Am J Roentgenol.

[5] Mostafavi MR, Ernst RD, Saltzman B (1998). Accurate determination of chemical composition of urinary calculi by spiral computerized tomography. J Urol.

[6] Saw KC, McAteer JA, Monga AG (2000). Helical CT of urinary calculi: effect of stone composition, stone size, and scan collimation. Am J Roentgenol.

[7] Nakada SY, Hoff DG, Attai S (2000). Determination of stone composition by noncontrast spiral computed tomography in the clinical setting. Urology.

[8] Motley G, Dalrymple N, Keesling C (2001). Hounsfield unit density in the determination of urinary stone composition. Urology.

[9] Deveci S, Coskun M, Tekin MI (2004). Spiral computed tomography: role in determination of chemical compositions of pure and mixed urinary stones -an in vitro study. Urology.

[10] Marchini GS, Remer EM, Gebreselassie S (2013). Stone characteristics on noncontrast computed tomography: establishing definitive patterns to discriminate calcium and uric acid compositions. Urology.

[11] Torricelli FC, Marchini GS, De S (2014). Predicting urinary stone composition based on single-energy noncontrast computed tomography: the challenge of cystine. Urology.

[12] Marchini GS, Gebreselassie S, Liu X (2013). Absolute Hounsfield unit measurement on noncontrast computed tomography cannot accurately predict struvite stone composition. J Endourol.

[13] Eisner BH, Kambadakone A, Monga M (2009). Computerized tomography magnified bone windows are superior to standard soft tissue windows for accurate measurement of stone size: an in vitro and clinical study. J Urol.

[14] Gucuk A, Uyeturk U, Ozturk U (2012). Does the Hounsfield unit value determined by computed tomography predict the outcome of percutaneous nephrolithotomy?. J Endourol.

[15] Tiselius HG, Andersson A (2003). Stone burden in an average Swedish population of stone formers requiring active stone removal: how can the stone size be estimated in the clinical routine?. Eur Urol.

[16] Frattini A, Barbieri A, Salsi P (2001). One shot: a novel method to dilate the nephrostomy access for percutaneous lithotripsy. J Endourol.

[17] De la Rosette JJ, Opondo D, Daels FP (2012). Categorisation of complications and validation of the Clavien score for percutaneous nephrolithotomy. Eur Urol.

[18] Turk C, Petrik A, Sarica K (2016). EAU guidelines on interventional treatment for Urolithiasis. Eur Urol.

[19] Manglaviti G, Tresoldi S, Guerrer CS (2011). In vivo evaluation of the chemical composition of urinary stones using dual-energy CT. Am J Roentgenol.

[20] De la Rosette J, Assimos D, Desai M (2011). The clinical research Office of the Endourological Society Percutaneous Nephrolithotomy Global Study: indications, complications, and outcomes in 5803 patients. J Endourol.

[21] Chua ME, Gatchalian GT, Corsino MV, Reyes BB (2012). Diagnostic utility of attenuation measurement (Hounsfield units) in computed tomography stonogram in predicting the radio-opacity of urinary calculi in plain abdominal radiographs. Int Urol Nephrol.

