# Supplementary material for: Clinical utility of computed tomography Hounsfield characterization for percutaneous nephrolithotomy: a cross-sectional study
Source: BMC Urol. 2017 Nov 16;17:104. doi: 10.1186/s12894-017-0296-1 (PMC5689164; doi:10.1186/s12894-017-0296-1)
Supplement: Supplementary file 1 — Patients’ epidemiologic and stone characteristics. (DOCX 13 kb) [file 12894_2017_296_MOESM1_ESM.docx]

**Supplementary Table 1** Patients’ epidemiologic and stone characteristics

|  | **Overall (%)** | **Calcium (%)** | **Uric acid**  **(%)** | **Cystine (%)** | **Struvite (%)** |
| --- | --- | --- | --- | --- | --- |
| Patients | 77 | 40 (52) | 26 (34) | 6 (8) | 5 (6) |
| Mean age | 57 | 58 | 61 | 32 | 59 |
| Female | 25 (33) | 12 (30) | 10 (38) | 0 | 3 (60) |
| Male | 52 (67) | 28 (70) | 16 (62) | 6 (100) | 2 (40) |
