# Supplementary material for: Clinical utility of computed tomography Hounsfield characterization for percutaneous nephrolithotomy: a cross-sectional study
Source: BMC Urol. 2017 Nov 16;17:104. doi: 10.1186/s12894-017-0296-1 (PMC5689164; doi:10.1186/s12894-017-0296-1)
Supplement: Supplementary file 2 — ROC curve of HUC on soft tissue window CT scan (HUCST) to discriminate between hypodense and hyperdense stones. Legend: HUC = HU at the center of the stone. (DOCX 16 kb) [file 12894_2017_296_MOESM2_ESM.docx]

**Supplementary Figure 1** ROC curve of HUC on soft tissue window CT scan (HUC_ST_) to discriminate between hypodense and hyperdense stones


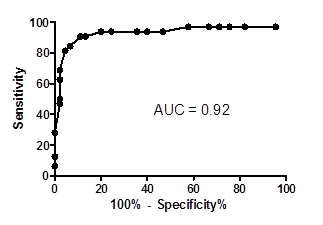


*Legend: HUC = HU at the center of the stone*
